# A single session of aerobic or resistance exercise modifies the endothelial progenitor cell levels in healthy subjects, but not in individuals with type 1 diabetes

**DOI:** 10.1186/1758-5996-7-S1-A251

**Published:** 2015-11-11

**Authors:** Beatriz D Schaan, Gustavo Waclawovsky, Daniel Umpierre, Franciele R Figueira, Eliandra S de Lima, Ana P Alegretti, Laiana Schneider, Ursula S Matte, Ticiana C Rodrigues

**Affiliations:** 1 Universidade Federal do Rio Grande do Sul/Hospital de Clínicas de Porto Alegre, Porto Alegre, Brazil

## Background

Endothelial progenitor cells (EPCs) from the bone marrow can regenerate the endothelium. In type 1 diabetes (T1D), EPCs are reduced as compared to healthy subjects, possibly accelerating endothelial dysfunction. Exercise mobilizes EPCs from bone marrow in some populations, but in T1D this was not previously studied.

## Objective

To evaluate the acute effect of aerobic (AE) and resistance (RE) exercise sessions on peripheral blood EPCs, blood flow (BF), vascular resistance (VR) and reactive hyperemia (RH) in patients with T1D.

## Materials and methods

We conducted a crossover randomized clinical trial, in which 14 men with T1D and 5 healthy controls were randomly assigned to a 40-min AE session (60% VO2peak) and a 40-min RE session (60% maximal load), 1 week apart. Venous blood was collected 10 min pre and post-exercise sessions to evaluate circulating EPCs (percentage of CD34+/KDR+/CD45dim of 200.000 mononuclear cells gated and analyzed by flow cytometry). Forearm BF and RH were evaluated by venous occlusion plethysmography before and after the sessions. Generalized Estimation Equation adjusted for baseline values was used.

## Results

Patients were 30.3±1.6 yrs-old, HbA1c 7.7±0.2%; controls were 26.8±2.3 yrs-old. Exercise did not change EPCs in T1D [AE (-5.075±0.250 vs. -5.303±0.250, P=0.102); RE (- 5.217±0.250 vs. -5.056±0.250, P=0.310)]; EPCs decreased after AE (-4.383±0.353 vs. - 4.854±0.353, P=0.017) and increased after RE (-5.270±0.353 vs. -4.629±0.353, P=0.004) in controls. Blood flow increased after RE in patients with diabetes (28.7±11.4%, P=0.009) and controls (41.7±18.5%, P=0.024). Reactive hyperemia was increased after AE (36.5±7.3%, P<0.001), and RE (42.0±10.0%, P<0.001) in patients with diabetes and controls (AE: 35.4±11.0%, P=0.001; RE: 74.3±33.0%, P=0.005).

## Conclusions

Despite the increases in RH in all subjects after both exercise sessions, EPCs were only influenced by exercise in controls. The unchanged number of EPCs in diabetes after both exercise protocols might indicate a blunted endothelium regenerating capacity, revealing a very early deterioration of the functional arterial characteristics not disclosed by only evaluating vascular functional variables.

